# Nanoplate Digital PCR for Identification of α^0^-Thalassemia (SEA Deletion): Carrier Screening and Possible Application to Prenatal Diagnosis

**DOI:** 10.3390/ijms27177846

**Published:** 2026-09-02

**Authors:** Apisit Pattrakorn, Supawadee Yamsri, Attawut Chaibunruang, Anupong Pansuwan, Wanicha Tepakhan, Kritsada Singha, Supan Fucharoen, Hataichanok Srivorakun

**Affiliations:** 1 Medical Sciences Program, Graduate School, Khon Kaen University, Khon Kaen 40002, Thailand; apisit_pattrakorn@kkumail.com; 2 Centre for Research and Development of Medical Diagnostic Laboratories, Faculty of Associated Medical Sciences, Khon Kaen University, Khon Kaen 40002, Thailand; supawadee@kku.ac.th (S.Y.); attach@kku.ac.th (A.C.); anuppa@kku.ac.th (A.P.); wanite@kku.ac.th (W.T.); kritsada.si@msu.ac.th (K.S.); supan@kku.ac.th (S.F.); 3 Faculty of Medicine, Mahasarakham University, Mahasarakham 44000, Thailand

**Keywords:** α^0^-thalassemia, Southeast Asian deletion, digital PCR, hemoglobin E, carrier screening, prenatal diagnosis

## Abstract

The Southeast Asian deletion (--^SEA^), the most prevalent α^0^-thalassemia mutation in Southeast Asia, is a major target of regional thalassemia prevention programs. Coinheritance of α^0^-thalassemia with other hemoglobinopathies, particularly hemoglobin E (Hb E), complicates routine hematological screening and requires reliable molecular confirmation. This study developed and validated a duplex nanoplate digital polymerase chain reaction (dPCR) assay for simultaneous identification of the wild-type α-globin and --^SEA^ deletion alleles in a single reaction. The assay was established on the QIAcuity nanoplate platform and evaluated using 171 blinded leftover DNA specimens from individuals with Hb E. Diagnostic performance was compared with conventional gap-PCR. Distinct fluorescence amplitude patterns enabled discrimination among normal, heterozygous, and homozygous --^SEA^ genotypes in a single closed tube. Among the 171 specimens, 70 were identified as --^SEA^ carriers and 101 as non-carriers, showing 100% concordance with gap-PCR. All subjects with Hb E + A_2_ levels ≥ 25% were negative for the --^SEA^ deletion, whereas 56.0% of those with levels < 25% carried the deletion. In the representative prenatal case, duplex dPCR accurately identified parental carrier status and a homozygous --^SEA^ fetus. This assay provides an accurate and practical molecular confirmatory assay for thalassemia screening and prenatal diagnosis in Southeast Asia.

## 1. Introduction

α-thalassemia is one of the most common inherited hemoglobin disorders worldwide, with the highest prevalence occurring in Southeast Asia, where carrier frequencies may exceed about 30–40% [1,2,3]. In northeast Thailand, the prevalence of α-thalassemia is around 30%, comprising about 5–6% α^0^-thalassemia and 25–30% α^+^-thalassemia [4]. α^0^-Thalassemia is caused by a deletion of two α-globin genes (--/αα), which in the homozygous state can lead to a fatal condition known as Hb Bart’s hydrops fetalis. The disease is almost invariably fatal in utero or shortly after birth and is associated with significant maternal complications [5]. Among the deletional forms, α^0^-thalassemia (SEA deletion) is the predominant α^0^-thalassemia mutation in Southeast Asia, including Northeast Thailand, with carriers accounting for 3.1–13.6% [6,7]. Consequently, α^0^-thalassemia (SEA deletion) is a target for a prevention and control program in the region [4,8].

Hemoglobin E (Hb E), a β-thalassemic hemoglobin variant, is among the most common hemoglobinopathies in Southeast Asia. A micro-mapping survey in northeast Thailand has revealed a carrier frequency of 41.7% [4]. Co-inheritance of Hb E and α-thalassemia is therefore frequently encountered in routine clinical practice in the region. In northeastern Thailand, it was demonstrated that double heterozygosity for Hb E and α^0^-thalassemia (--^SEA^) occurred in approximately 1.4–10.4% [6,9].

Coinheritance of Hb E and α-thalassemia modifies hematological characteristics, leading to lower Hb E percentages and more pronounced microcytosis and hypochromia than that of pure Hb E carriers [1,10]. As a consequence, conventional hematological screening based on mean corpuscular volume (MCV), mean corpuscular hemoglobin (MCH), and Hb E percentage alone cannot reliably distinguish Hb E carriers with concomitant α^0^-thalassemia from those without α-globin gene deletions [11,12,13,14]. Molecular confirmation is therefore essential for accurate carrier diagnosis, genetic counseling, and risk assessment.

Several molecular techniques have been developed for detecting the --^SEA^ deletion, including gap-polymerase chain reaction (gap-PCR) [15], quantitative real-time PCR (qPCR) [16], multiplex ligation-dependent probe amplification (MLPA) [17], and next-generation sequencing (NGS) [18]. Although these methods demonstrate excellent analytical performance, they usually require multiple post-amplification procedures and multiple workflows, increasing laboratory workload, turnaround time, reagent consumption, and operational costs [19].

Digital polymerase chain reaction (dPCR) has emerged as a third-generation PCR technology that partitions a PCR reaction into thousands of independent microreactions, enabling absolute nucleic acid quantification using Poisson statistics without requiring standard curves. Compared with conventional PCR and qPCR, dPCR provides superior analytical precision, greater tolerance to PCR inhibitors, and improved performance for copy number variation and structural variant analysis [20,21,22].

Several studies have demonstrated the feasibility of droplet-based digital PCR for detecting the --^SEA^ deletion [23,24,25]. The clinical implementation of nanoplate-based digital PCR for non-invasive prenatal diagnosis of thalassemia has been reported [26]; however, few studies have evaluated this technology for screening of α^0^-thalassemia in Hb E carriers, a clinically important population in whom concomitant α-thalassemia frequently complicates interpretation of routine screening results.

In the present study, we developed and validated a QIAcuity nanoplate-based duplex digital PCR assay for the simultaneous detection of the wild-type and SEA deletion alleles in a single reaction and evaluated its clinical performance for screening α^0^-thalassemia in Hb E carriers.

## 2. Results

**Figure 1:**
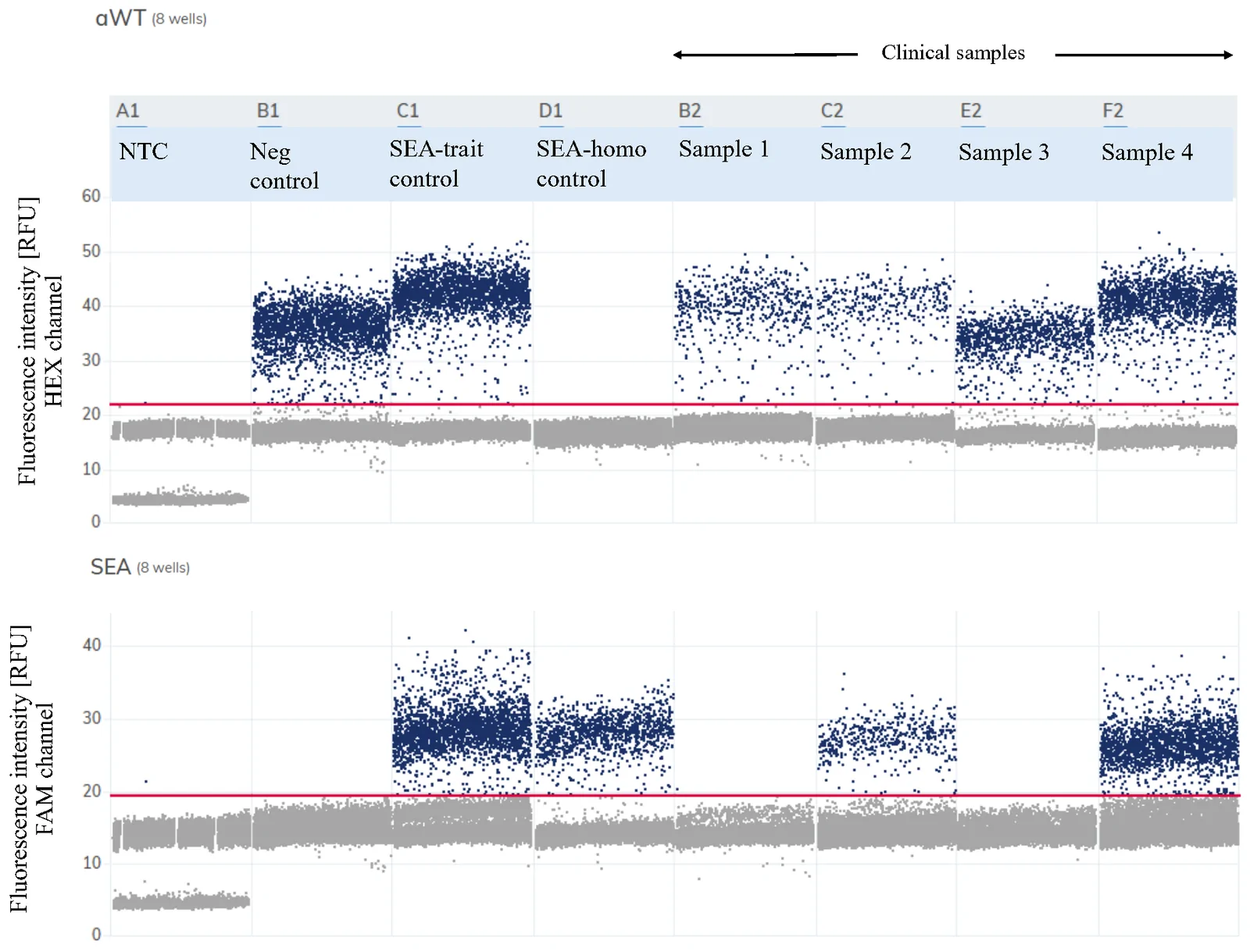
Fluorescence amplitude plots of the duplex nanoplate digital PCR assay for detection of the α^0^-thalassemia Southeast Asian (--^SEA^) deletion. Representative one-dimensional fluorescence amplitude plots of the HEX channel (upper) for wild-type α-globin allele detection and the FAM channel (lower) for α^0^-thalassemia (--^SEA^ deletion) allele detection. The A1–D1 show the fluorescence amplitude plots of the no-template control (NTC), wild-type control (Neg-control), --^SEA^ carrier control (SEA-trait control), and homozygous --^SEA^ control (Homo-SEA control), respectively. In clinical specimens from non-α^0^-thalassemia (SEA deletion), fluorescence was observed only in the HEX channel (B2 and E2). The fluorescence intensities of both the HEX and FAM channels were observed in SEA deletion carriers (C2 and F2), corresponding to the control. The fluorescence intensity was observed only in the FAM channel for the homozygous α^0^-thalassemia (SEA deletion) genotype, as in the SEA-homo control. The red line was a threshold line. Blue and gray dots represent fluorescence-positive and fluorescence-negative partitions, respectively. RFU = Relative Fluorescence Units.

Figure 1 shows the developed duplex nanoplate dPCR results: a simple one-dimensional fluorescence amplitude plot of a dual-fluorescence probe design for the simultaneous detection of the α^0^-thalassemia (SEA deletion) allele and the wild-type α-globin allele. The fluorescence intensity of the wild-type allele-specific probe (HEX channel) and the SEA deletion allele-specific probe (FAM channel) of NTC, negative control, SEA-trait control, and homo-SEA control were shown in Figure 1, A1–D1, respectively. In non-α^0^-thalassemia (SEA deletion), the fluorescence intensity was observed only in the HEX channel (Figure 1, B2 and E2), consistent with the negative control. The fluorescence intensity of both HEX and FAM channels was observed in SEA deletion carriers (Figure 1, C2 and F2), consistent with the SEA-trait control. For the homozygous α^0^-thalassemia (SEA deletion) genotype (SEA-homo control), fluorescence was observed only in the FAM channel. The one-dimensional fluorescence plots clearly discriminated normal, heterozygous, and homozygous --^SEA^ genotypes without ambiguous signal interpretation, highlighting the robustness of the duplex assay for molecular diagnosis.

To validate the developed nanoplate duplex digital PCR assay for detecting α^0^-thalassemia (SEA deletion), a total of 171 leftover DNA specimens from Hb E carriers were tested. The results of this dPCR assay were compared with those obtained from the conventional gap-PCR method, which served as the gold standard. Of these 171 subjects, 70 were identified as carriers of α^0^-thalassemia (SEA deletion); the remaining 101 subjects were negative. The results are 100% concordant and 100% accurate with the conventional gap-PCR method.

The hematological data of these 171 validated specimens are shown in Table 1. Based on the (Hb E + A_2_) levels, subjects were classified into two groups: Hb E trait (Hb E + A_2_ ≥ 25%; *n* = 46) and samples with Hb E + A_2_ < 25% (*n* = 125). Among the latter group, 70 subjects (56.0%) were positive for the --^SEA^ deletion, whereas 55 subjects (44.0%) were negative by duplex digital PCR. In contrast, none of the individuals with Hb E + A_2_ ≥ 25% carried the --^SEA^ deletion. Individuals harboring the --^SEA^ deletion exhibited more microcytic and hypochromic red cell indices than those without the deletion, with the median MCV being 66.0 fL vs. 72.2 fL, and MCH was 21.2 vs. 23.2 pg, respectively. The --^SEA^-positive group also showed lower median hemoglobin (11.2 vs. 12.5 g/dL) and hematocrit (35.0% vs. 38.8%).

**Figure 2:**
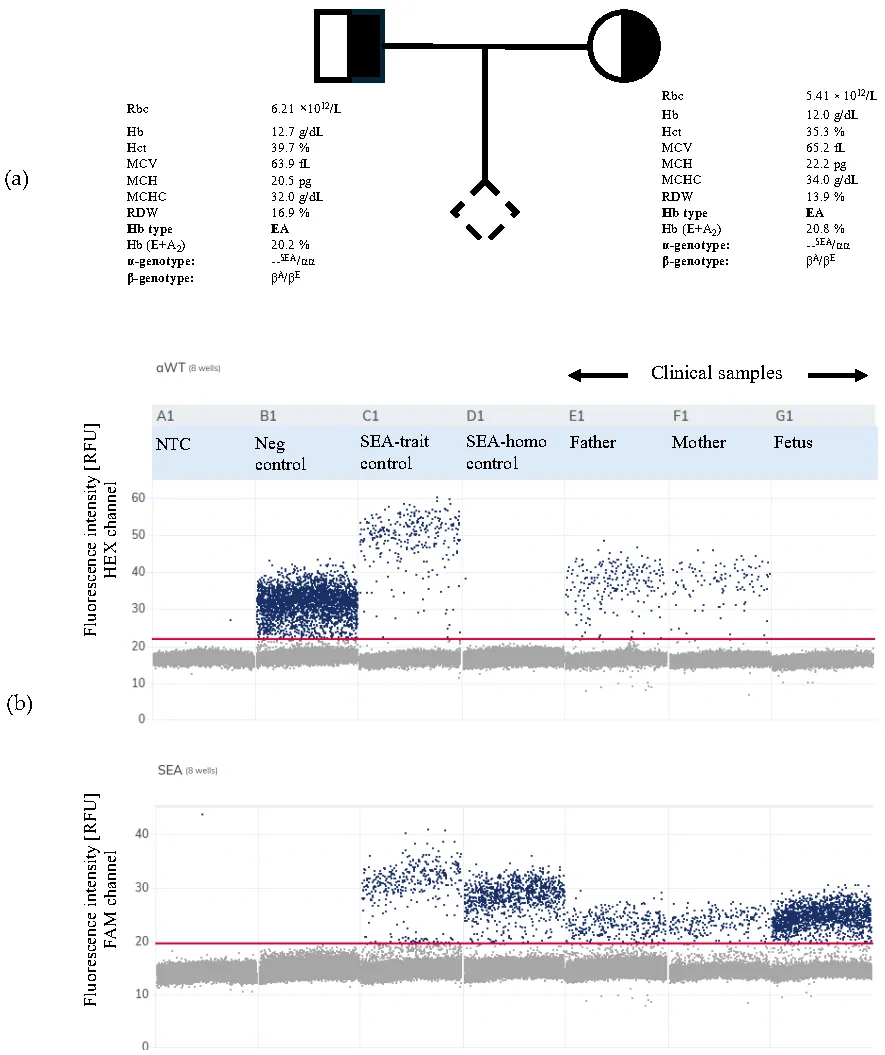
Applications of the developed duplex nanoplate dPCR to prenatal diagnosis in couples at risk of having severe thalassemia in the fetuses. (**a**) Based on hematological and Hb analyses, both father and mother were heterozygous for Hb E, with or without α^0^-thalassemia, requiring further DNA analysis for α^0^-thalassemia to confirm a true risk couple for severe thalassemia. (**b**) The simple one-dimensional plot of duplex dPCR for α^0^-thalassemia (SEA deletion) detection in the family. Both father and mother were heterozygous Hb E with α^0^-thalassemia (SEA deletion) (E1 and F1, respectively). Therefore, the couple is at risk of 25% of having a fetus with the most severe homozygous α^0^-thalassemia (Hb Bart’s hydrops fetalis). Prenatal diagnosis with a fetal DNA sample extracted from amniotic fluid showed a homozygous state of α^0^-thalassemia (SEA deletion) (G1) corresponding with routinely conventional gap-PCR.

To further demonstrate the clinical applicability of the developed duplex digital PCR assay, a representative family at risk of severe thalassemia was investigated, as shown in Figure 2. In Figure 2a, based on hematological findings, both parents showed microcytic hypochromic erythrocytes with Hb E + A_2_ levels less than 25%, possibly co-inheritance with α-thalassemia, requiring molecular characterization. In Figure 2b, duplex digital PCR identified, as expected, that both the father and the mother carried the --^SEA^ deletion, thereby confirming the couple to be at risk of conceiving a fetus with homozygous α^0^-thalassemia (Hb Bart’s hydrops fetalis). Prenatal diagnosis was performed on amniotic fluid, which demonstrated a complete absence of the wild-type allele and exclusive detection of the --^SEA^ deletion allele, indicating homozygous α^0^-thalassemia (--^SEA^/--^SEA^). The digital PCR result was fully concordant with the conventional gap-PCR reference method.

## 3. Discussion

The present study describes the development and clinical validation of a duplex nanoplate digital PCR (dPCR) assay for simultaneous detection of the wild-type α-globin allele and the α^0^-thalassemia (SEA deletion) allele in a single reaction. The assay demonstrated complete concordance with the conventional gap-PCR method while providing a simplified closed-tube workflow that enables direct genotype discrimination through fluorescence signal detection.

The one-dimensional fluorescence amplitude plots generated distinct fluorescence signatures corresponding to normal, heterozygous, and homozygous --^SEA^ genotypes without overlapping signal patterns. This clear separation indicates high analytical specificity of both allele-specific hydrolysis probes and demonstrates that simultaneous amplification of two targets did not compromise assay performance. The endpoint fluorescence-based interpretation also minimizes ambiguity associated with amplification kinetics and provides straightforward genotype assignment. These findings are consistent with the fundamental principle of digital PCR, in which thousands of independent partitions allow endpoint detection of individual target molecules with high analytical precision while reducing competition between amplification targets in multiplex assays [20,21,22].

Gap-PCR remains the standard method for detecting common α-globin gene deletions because of its simplicity and diagnostic reliability; however, it requires post-amplification agarose gel electrophoresis, which increases turnaround time and the risk of amplicon contamination. Quantitative real-time PCR eliminates the need for electrophoresis but relies on amplification efficiency and cycle threshold (Ct) values for relative quantification, making assay performance susceptible to variations in DNA quality and PCR efficiency [19]. In contrast, digital PCR relies on endpoint fluorescence measurement and Poisson statistics to provide absolute molecular detection without requiring standard curves or Ct-based interpretation [20]. The nanoplate-based QIAcuity platform further integrates reaction partitioning, PCR amplification, fluorescence imaging, and automated data analysis within a single instrument [27], thereby simplifying laboratory workflow and reducing hands-on time. These characteristics make the assay suitable for routine molecular diagnostics in clinical laboratories. Compared with gap-PCR, the principal practical advantage of the present assay is that genotype discrimination is achieved from endpoint fluorescence in a closed-tube reaction, avoiding post-amplification agarose gel electrophoresis and reducing opportunities for amplicon contamination. The nanoplate platform also integrates partitioning, amplification, fluorescence imaging, and automated analysis within a single instrument, simplifying the overall workflow. In addition, the duplex nanoplate dPCR assay provided a shorter overall turnaround time, requiring approximately 2.5 h from reaction preparation to result interpretation, compared with approximately 3.5 h for conventional gap-PCR, which includes PCR amplification followed by agarose gel electrophoresis and result interpretation.

Clinical validation using 171 Hb E carriers demonstrated complete agreement between the developed duplex dPCR assay and the reference gap-PCR method, yielding 100% concordance for identifying the --^SEA^ deletion. Although previous studies [23,24,25] have demonstrated the feasibility of droplet digital PCR for detecting the Southeast Asian deletion, most were performed using droplet-based platforms or focused primarily on analytical validation. The present study demonstrates that comparable diagnostic performance can be achieved using a nanoplate partitioning platform while simultaneously validating the assay in a clinically relevant cohort of Hb E carriers, a population frequently encountered in Southeast Asia, where both Hb E and α^0^-thalassemia are highly prevalent.

The hematological characteristics of the validation cohort further demonstrated the well-recognized modifying effect of α^0^-thalassemia on the hematological phenotype of Hb E carriers. In the present study, all individuals with Hb E + A_2_ levels ≥ 25% were negative for the --^SEA^ deletion, whereas around half of the subjects with Hb E + A_2_ levels < 25% carried the --^SEA^ deletion. This observation supports previous reports that coinheritance of α-thalassemia reduces Hb E synthesis, resulting in lower Hb E percentages than those observed in pure Hb E heterozygotes [9,10]. In individuals carrying the --^SEA^ deletion, reduced α-globin synthesis limits Hb E production, leading to a relative decrease in the proportion of Hb E detected by Hb analysis. Hb E carriers of the --^SEA^ deletion exhibited lower MCV, MCH, hemoglobin concentration, and hematocrit than non-carriers, consistent with the more pronounced microcytic and hypochromic phenotype associated with reduced α-globin synthesis. These findings agree with previous studies demonstrating that coinheritance of α-thalassemia modifies Hb E expression and exacerbates microcytosis because reduced α-globin availability limits Hb E tetramer formation [2,10]. Nevertheless, substantial overlap in hematological parameters between carriers and non-carriers indicates that hematological screening alone cannot reliably establish α^0^-thalassemia carrier status [12,13,14], particularly in populations where multiple hemoglobinopathies coexist. Molecular confirmation therefore remains essential for accurate diagnosis and genetic counseling.

**Figure 3:**
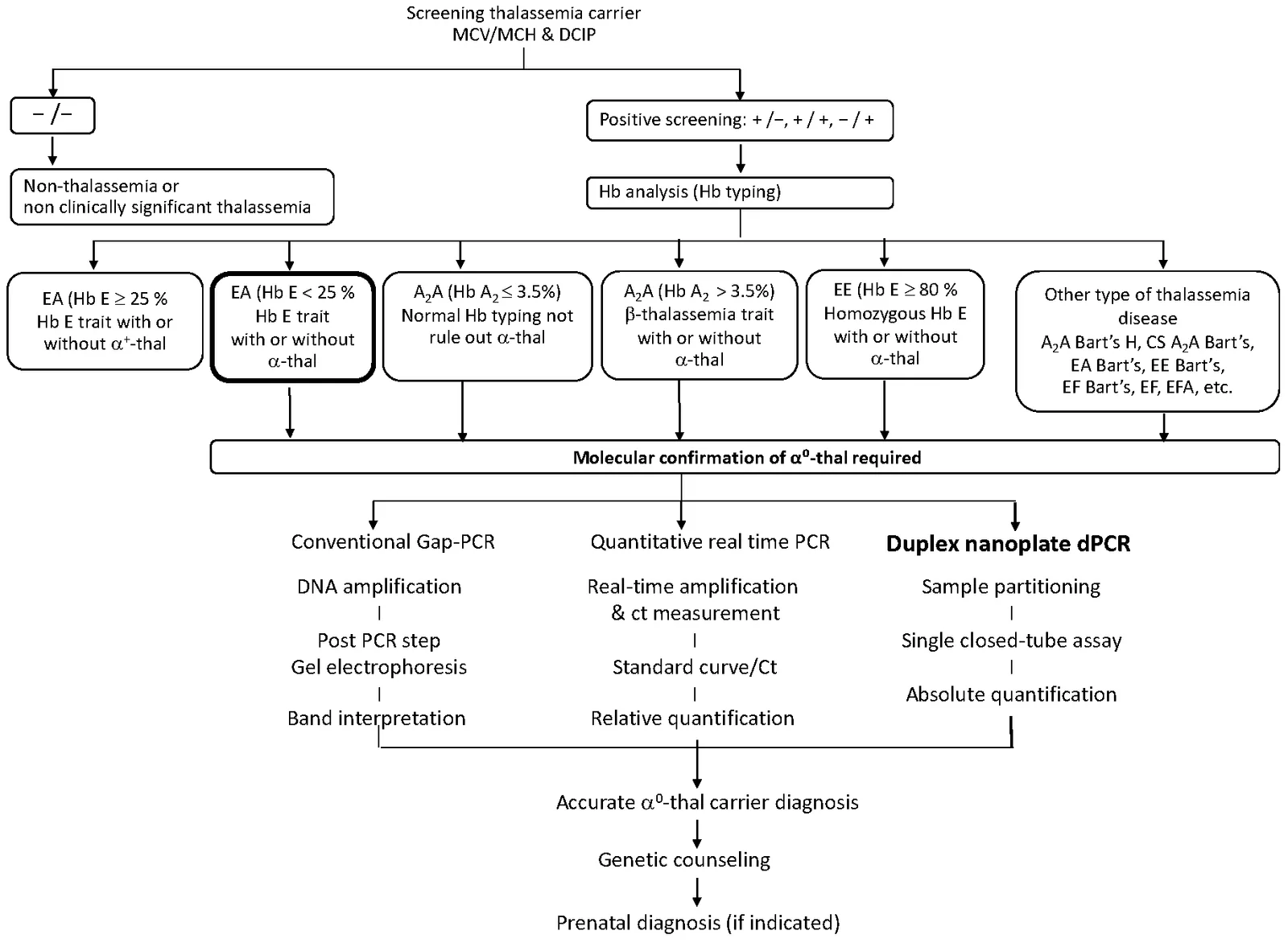
Proposed molecular diagnostic workflow for confirmation of α^0^-thalassemia (SEA deletion) using duplex nanoplate digital PCR in thalassemia screening programs. Proposed diagnostic workflow illustrating the integration of the duplex nanoplate digital PCR (dPCR) assay into routine thalassemia screening. Positive initial screening of an individual using MCV/MCH and the dichlorophenolindophenol (DCIP) test was followed by hemoglobin analysis. Individuals with hematological findings suggestive of α-thalassemia, particularly Hb E carriers with reduced Hb E percentages, undergo molecular confirmation for the α^0^-thalassemia Southeast Asian (--^SEA^) deletion. The workflow compares three molecular approaches: conventional gap-PCR, which requires post-amplification gel electrophoresis for genotype interpretation; quantitative real-time PCR, which relies on amplification kinetics and cycle threshold (Ct)-based relative quantification; and the duplex nanoplate dPCR assay developed in this study, which enables simultaneous detection of the wild-type α-globin allele and the --^SEA^ deletion allele in a single closed-tube reaction using endpoint fluorescence analysis and absolute molecular quantification. Accurate molecular diagnosis supports carrier identification, genetic counseling, and prenatal diagnosis for severe thalassemia syndrome.

The clinical implication of the present study is the application of duplex nanoplate dPCR to prenatal diagnosis. The representative family investigated in this study illustrates a common diagnostic scenario encountered in Southeast Asian thalassemia prevention programs. Both parents presented with hematological findings compatible with Hb E trait but required molecular testing to determine whether concomitant α^0^-thalassemia was present. The duplex dPCR assay rapidly confirmed that both parents carried the --^SEA^ deletion, thereby identifying a 25% risk of Hb Bart’s hydrops fetalis in each pregnancy. Analysis of fetal DNA obtained from amniotic fluid subsequently identified a homozygous --^SEA^ genotype, and the result was completely concordant with conventional gap-PCR. These findings demonstrate that the developed assay is applicable not only for carrier screening but also for prenatal diagnosis and reproductive risk assessment. The assay may be incorporated into existing thalassemia prevention and control programs as a confirmatory molecular test following routine hematological screening. As shown in Figure 3, following routine hematological screening, Hb E carriers with Hb E level < 25% undergo molecular testing to determine whether α^0^-thalassemia (--^SEA^ deletion) is coinherited [28]. Conventional gap-PCR and quantitative PCR are currently available molecular approaches, whereas the duplex nanoplate digital PCR assay developed in this study enables simultaneous detection of the wild-type α-globin allele and the --^SEA^ deletion allele in a single closed-tube reaction using endpoint fluorescence analysis.

The limit of detection (LoD) of the developed duplex nanoplate dPCR assay was evaluated using 10-fold serial dilutions of genomic DNA from an α^0^-thalassemia (--^SEA^ deletion) carrier. The LoD was established at 0.44 ng/reaction, at which the --^SEA^ deletion was consistently detected in all 20 replicates (20/20, 100%). This analytical sensitivity suggests the assay’s potential applicability to prenatal specimens with limited DNA availability. However, because the LoD was determined using diluted genomic DNA, further validation by actual low-input prenatal specimens is required to establish its clinical performance in prenatal diagnosis. Non-invasive prenatal diagnosis using cell-free fetal DNA in maternal plasma has also been investigated for thalassemia, including digital PCR-based approaches [26]. However, the applicability of the present assay to cell-free DNA requires further validation, as the current LoD was established using genomic DNA. Similarly, the suitability of the present assay for preimplantation genetic diagnosis using exhausted embryo culture medium remains to be investigated further.

In conclusion, the duplex QIAcuity nanoplate digital PCR assay provides a rapid, accurate, and robust method for simultaneous detection of the wild-type α-globin allele and the --^SEA^ deletion allele in a single closed-tube reaction. Its excellent diagnostic performance, simplified workflow, and demonstrated applicability in carrier screening and prenatal diagnosis support its potential implementation as a molecular confirmatory assay within thalassemia prevention and control programs in Southeast Asia.

## 4. Materials and Methods

### 4.1. Study Design and Samples

In this study, all leftover DNA specimens were recruited from the Thalassemia Service Unit (TSU), Center for Research and Development of Medical Diagnostic Laboratories (CMDL), Khon Kaen University, Khon Kaen, Thailand.

Initial development of digital PCR (dPCR) assays was done using leftover DNA specimens with known α^0^-thalassemia (SEA deletions) genotype. Prospective validation in a blinded trial was assessed using a total of 171 leftover DNA specimens characterized as Hb E trait by Hb analysis. Those prospective samples were positive in the initial screening using reduced MCV and MCH and the dichlorophenolindophenol (DCIP) test for Hb E, and were referred to our thalassemia service unit for further Hb and DNA analysis. The hematological parameters obtained using a hematology analyzer (Sysmex, Kobe, Japan) were recorded. Hb analysis was performed routinely using a capillary electrophoresis system (Capillarys 2 Flex Piercing, Sebia, Lisses, France). The dPCR results were compared with those obtained with the standard gap-PCR method used in our routine setting [15].

### 4.2. Nanoplate Digital PCR for α-Thalassemia South-East Asian-Type Deletion Diagnosis

We developed a duplex digital PCR assay for simultaneous detection of the wild-type α-globin gene and α^0^-thalassemia (SEA deletion) alleles on the QIAcuity nanoplate platform. The dPCR assay was performed using a fully automated instrument, the QIAcuity Digital PCR System (Qiagen; Hilden, Germany). Only the plate preparation was done manually before starting the run. In every analysis, the nanoplate includes one well of each sample and each well of no-template control (NTC), non-α^0^-thalassemia (SEA deletion) control (neg control), positive heterozygous α^0^-thalassemia (SEA deletion) control (SEA-trait control), and positive homozygous α^0^-thalassemia (SEA deletion) control (SEA-homo control).

**Figure 4:**
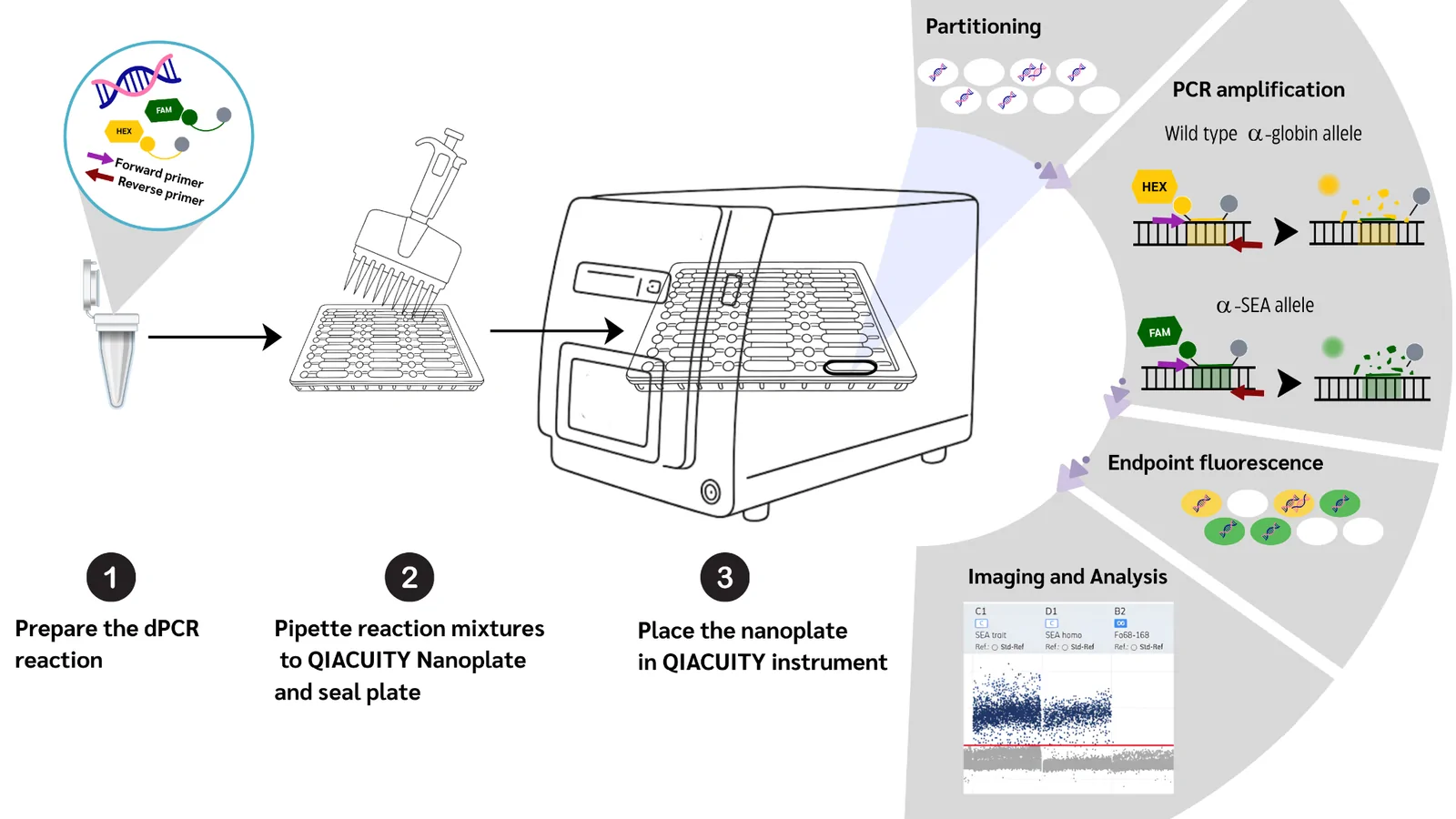
Workflow of the duplex nanoplate digital PCR assay for detection of the α^0^-thalassemia Southeast Asian (--^SEA^) deletion. The workflow illustrates the major steps of the assay, including preparation of the duplex dPCR reaction mixture, loading and partitioning of samples in the nanoplate, PCR amplification, fluorescence imaging, and automated data analysis. Black arrows indicate the sequential steps of the workflow. Purple and red arrows represent the forward and reverse primers, respectively. Yellow and green symbols represent the HEX-labeled probe targeting the wild-type α-globin allele and the FAM-labeled probe targeting the --^SEA^ deletion allele, respectively. Yellow and green partitions indicate positive fluorescence signals in the corresponding channels, whereas white partitions indicate no detectable fluorescence. Genotypes are determined from the combined HEX- and FAM-channel fluorescence patterns.

**Figure 5:**
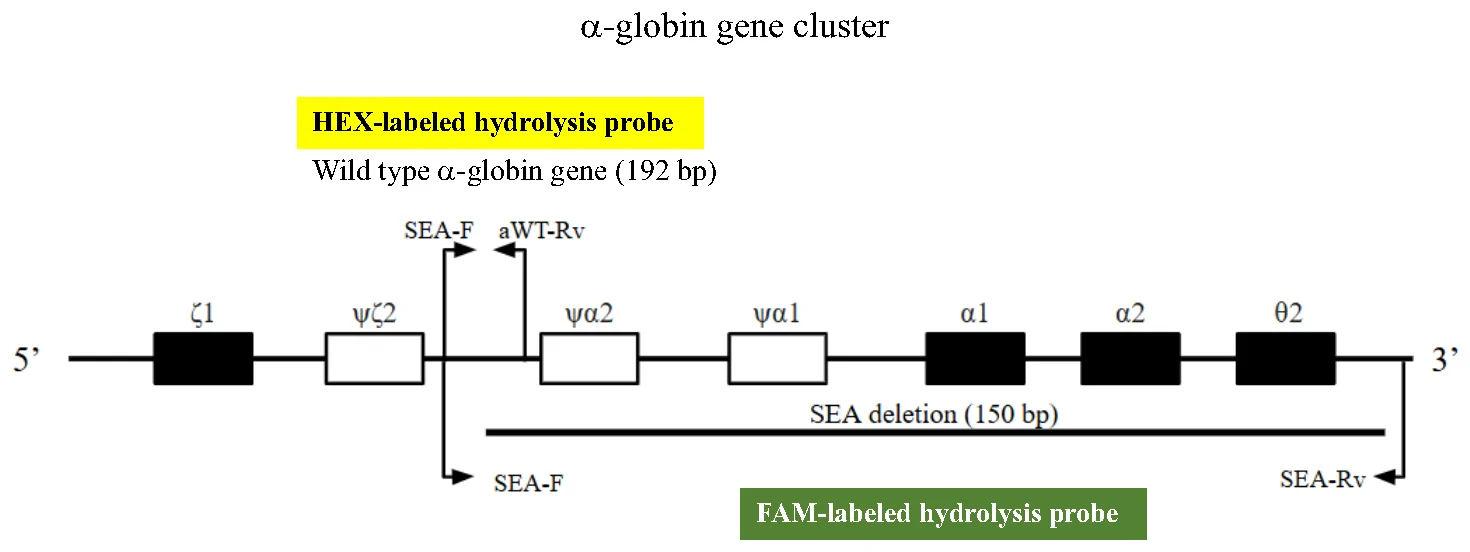
Locations and orientations of primers and probes for the identification of α^0^-thalassemia (SEA deletion) by the developed duplex nanoplate digital PCR assay. Schematic illustration showing the locations of the allele-specific primers and hydrolysis probes used in the duplex nanoplate digital PCR assay. One primer/probe set was designed to specifically amplify the wild-type α-globin allele (SEA-F and aWT-Rv) and was detected using a HEX-labeled hydrolysis probe, whereas the second primer/probe set targeted the unique --^SEA^ deletion breakpoint (SEA-F and SEA-Rv) and was detected using a FAM-labeled hydrolysis probe. Simultaneous amplification of both targets in a single reaction.

The workflow of the developed duplex nanoplate digital PCR assay is shown in Figure 4. All primers and probes were designed using the Primer3Plus version 3.3.0 program according to the guidelines provided by QIAGEN. The details of the primers and probes are shown in Table 2. The locations and orientations of the primers are demonstrated in Figure 5. A 150 bp specific fragment for α^0^-thalassemia (SEA deletion) that contained a specific sequence for the SEA probe was generated using the SEA-Forward primer (SEA-F) and SEA-Reverse primer (SEA-Rv). A 192 bp normal fragment of the α-globin gene that was generated using SEA-F and alpha wild-type reverse primer (aWT-Rv) contained a specific sequence for the alpha wild-type probe (aWT-probe). Each reaction mixture (40 μL) contained 10 µL of 4X QIAcuity Probe PCR master mix, 6.4 µL of SEA-F, each 3.2 µL of SEA-Rv, aWT-Rv, and aWT-Probe, 1.6 µL of SEA-Probe, 2 µL of DNA template (approximately 2.16 μg), and 10.4 μL of nuclease-free water. The reaction mixtures were loaded into a 26k nanoplate 24-well plate (Qiagen). The nanoplate was transferred into the QIAcuity One Digital PCR System (QIAGEN). The PCR cycling profile began with 2 min at 95 °C, followed by 40 cycles of 15 s at 95 °C, 30 s at 60.7 °C, and 30 s at 72 °C. A total of 20,000–25,000 partitions per reaction were created in the nanoplate. Fluorescence signals in the partitions were measured using the QIAcuity One Digital PCR System (QIAGEN). They were analyzed using QIAcuity Software Suite version 3.1.0.0 (QIAGEN).

### 4.3. Interpretation of Digital PCR Results

The well with more than 10,000 valid partitions was analyzed. Digital PCR data were analyzed using QIAcuity Software Suite version 3.1.0.0 (QIAGEN). Fluorescence thresholds were established for each channel based on non-template control (NTC), positive control, and negative control. Partitions with fluorescence amplitudes above the adjusted threshold were classified as positive, whereas those below the threshold were classified as negative. In the developed dPCR assay, HEX channel fluorescence indicated the presence of the wild-type α-globin allele, while the fluorescence of the FAM channel indicated the presence of the α^0^-thalassemia (SEA deletion) allele (Figure 1).

### 4.4. Statistical Analysis

The statistical analysis was performed using SPSS Statistics v.29. Hematological values and Hb profiles among the prospective sample were described by median, minimum, and maximum values. Percentage of accuracy and concordance were calculated and compared with the results obtained from the conventional gap-PCR, which served as the gold standard.

## Figures and Tables

**Table 1:** Hematological data of 171 validated EA-typing samples with positive and negative --^SEA^ gene in duplex digital PCR assays for α^0^-thalassemia (SEA deletion).

Hb Typing	--^SEA^ Gene	*n*	Hb E + A_2_ (%)	Hb F (%)	RBC (×10^12^/L)	Hb (g/dL)	Hct (%)	MCV (fl)	MCH (pg)	MCHC (g/dL)	RDW-CV (%)
EA; %Hb E + A_2_ ≥ 25	Positive	0	-	-	-	-	-	-	-	-	-
	Negative	46	28.0 (25.3–30.1)	0.0 (0.0–2.6)	5.0 (3.6–6.6)	12.3 (10.1–15.6)	38.0 (30.8–47.4)	76.8 (69.1–87.4)	25.0 (22.9–28.8)	32.7 (30.7–34.5)	13.9 (11.6–17.8)
EA; %Hb E + A_2_ < 25	Positive	70	19.7 (13.7–22.5)	0.0 (0.0–4.8)	5.5 (3.9–7.2)	11.2 (7.0–14.2)	35.0 (24.6–45.9)	66.0 (42.7–74.8)	21.2 (13.1–21.4)	32.0 (18.8–34.3)	15.8 (12.2–27.4)
	Negative	55	23.0 (14.5–24.9)	0.0 (0.0–3.2)	5.4 (4.0–6.9)	12.5 (7.7–16.1)	38.8 (25.0–52.0)	72.2 (44.5–87.8)	23.2 (13.6–29.6)	32.6 (28.5–33.8)	14.2 (11.4–21.0)
	Total	171									

Values are presented as median (Minimum–Maximum).

**Table 2:** List of primers and probe sequences used for detection of α^0^-thalassemia (SEA deletion) and the wild-type allele.

Primer/Probe	Sequence (5′ → 3′)
SEA-F	5′-GGCCTAAAACCACAGAGT-3′
SEA-Rv	5′-CCAGAAGCGAGTGTGTGGAA-3′
SEA-probe	5′-/56-FAM/TTCACTTGG/ZEN/AGGCTGGGGCA/3IABkFQ/-3′
aWT-F	5′-ACTCAAGGACCCCAGGAGAC-3′
aWT-probe	5′-/5SUN/AGGTTCTAG/ZEN/CCCCTGAGCAC/3IABkFQ/-3′

## Data Availability

All relevant data are within the paper. Further inquiries can be directed to the corresponding author.
